# 3D Spatiotemporal Activation Mapping of Cardiac Organoids Using Conformal Shell Microelectrode Arrays (MEAs)

**DOI:** 10.21203/rs.3.rs-5939602/v1

**Published:** 2025-02-07

**Authors:** Soo Jin Choi, Zhaoyu Liu, Feiyu Yang, Hanwen Wang, Derosh George, David H. Gracias, Deok-Ho Kim

**Affiliations:** 1 Department of Chemical and Biomolecular Engineering, Johns Hopkins University, Baltimore, MD 21218; 2 Department of Biomedical Engineering, Johns Hopkins University, Baltimore, MD 21218; 3 Department of Materials Science and Engineering, Johns Hopkins University, Baltimore, MD, USA.; 4 Department of Chemistry, Johns Hopkins University, Baltimore, MD 21218; 5 Laboratory for Computational Sensing and Robotics (LCSR), Johns Hopkins University, Baltimore, MD 21218; 6 Sidney Kimmel Comprehensive Cancer Center, Johns Hopkins School of Medicine, Baltimore, MD, 21205; 7 Center for Microphysiological Systems, Johns Hopkins University, Baltimore, MD 21205; 8 Department of Oncology, Johns Hopkins University School of Medicine, Baltimore, MD 21205; 9 Department of Medicine, Johns Hopkins University, Baltimore, MD 21205; 10 Institute for NanoBioTechnology, Johns Hopkins University, Baltimore, MD 21218; 11 Department of Mechanical Engineering, Johns Hopkins University, Baltimore, MD 21218

## Abstract

Cardiac organoids have emerged as transformative models for investigating cardiogenesis and cardiac diseases. While traditional 2D microelectrode arrays (MEAs) have been used to assess the functionality of cardiac organoids, they are limited to electrophysiological measurements from a single plane and do not capture the 3D propagation of electrical signals. Here, we present a programmable, shape-adaptive shell MEA designed to map the electrical activity across the entire surface of cardiac organoids. These shell MEAs are fabricated on-chip, with tunable dimensions and electrode layout, enabling precise encapsulation of spherical organoids. Using shell MEAs, we generated 3D isochrone maps with conduction velocity vectors, revealing the speed and trajectory of electrical signal propagation in spontaneously beating cardiac organoids. The optical transparency of the shell MEAs allowed for simultaneous calcium imaging, validating the electrophysiological propagation pattern. To demonstrate their utility in cardiotoxicity screening, we monitored the electrophysiological changes of organoids treated with isoproterenol and E-4031 over nine days. We anticipate that shell MEAs, combined with spatiotemporal mapping, can significantly advance the development of spatially organized cardiac organoids, structural disease models, and high-throughput drug screening platforms.

## Introduction

Activation mapping plays a critical role in diagnosing and treating arrhythmias^1^. In clinical practice, catheters with electrodes are inserted inside a patient’s heart to measure electrical signals, which are used to create a detailed map of the localized electrical activity^2^. These voltage and activation maps reveal key conduction pathways and identify arrhythmogenic foci, which can be targeted and ablated during catheter ablation procedures to treat arrhythmias^3^. Human induced pluripotent stem cell (iPSC)-derived cardiac organoids have significantly advanced our understanding of cardiogenesis and cardiovascular diseases due to their capacity to recapitulate the cytoarchitecture and cellular composition of native hearts^4–6^. In the context of arrhythmias, cardiac organoids have been found to generate spontaneous and elicited action potentials with higher conduction velocities compared to two-dimensional (2D) models^4^. Moreover, these three-dimensional (3D) cardiac models allow the investigation of multicellular, tissue-scale arrhythmic events, such as myocardial interstitial fibrosis, in addition to cellular-level arrhythmic triggers, such as afterdepolarizations and calcium waves^7^. However, the lack of organoid-interfacing analytical tools that can detect electrical signals across whole organoids is a significant bottleneck for leveraging these 3D tissue models for spatiotemporally resolved studies.

Optical imaging-based techniques that utilize calcium indicators or voltage-sensitive dyes face limitations for long-term recording due to the decline of fluorescent signals and the challenge of visualizing the electrical propagation along the vertical axis, owing to the narrow vertical focal plane^2,8–10^. Electrophysiology (EP) techniques, such as patch clamps and 2D microelectrode arrays (MEAs), can detect electrical signals with high temporal resolution (>10 kHz)^11^. However, organoids must be dissociated into single cells for patch clamp analysis, limiting their use to end-point measurements^12^. 2D MEAs offer a less invasive approach to assess cardiac function *in situ* but are constrained to measuring signals from only the bottom plane of organoids and often require adhesive coatings to enhance cell attachment for adequate signal-to-noise ratios (SNR), which compromises the 3D physiological structure of the organoid^13^. Importantly, both patch clamp and 2D MEAs cannot characterize 3D wavefront propagation—an important parameter for assessing the health and function of the myocardial substrate^14,15^.

Researchers have aimed to address these limitations by developing unconventional 3D MEAs for engineered tissues. Probe microelectrodes have been inserted in brain and cardiac organoids to record intraorganoid electrophysiology^16–19^. Planar mesh electrodes have been used to culture neonatal rat ventricular cardiomyocytes, which were folded into multilayered tissue to map 3D action potential propagation^20,21^. Other researchers have suspended neural and cardiac organoids on deformable mesh MEAs to record electrophysiological data^22–25^. Stretchable mesh electrodes have also been embedded within cardiac organoids and microtissues during morphogenesis^26–28^. Self-rolling biosensor arrays have been used to wrap around cardiac spheroids and detect their mean conduction velocity from a 2D isochrone map^29^. Many of these devices and other elastomer-based MEAs require micromanipulators to place the organoids in precise positions, highlighting the challenges of creating high-throughput 3D MEAs^30–33^. To our knowledge, to date, no MEA has demonstrated the ability to map electrical propagation across entire 3D surfaces of cardiac organoids while preserving their natural self-assembled architecture^34^.

In this study, we present a novel 3D electrophysiology recording platform that facilitates the visualization of spontaneous activations and arrhythmogenic mechanisms in organoids. We build on prior studies of shell MEAs for brain organoids^35^ with significant advances: (a) the use of a transparent substrate for compatibility with optical imaging, (b) a refined MEA design with 16 electrodes accommodating a wider range of organoid sizes, and (c) a comprehensive 3D spatiotemporal mapping scheme. By leveraging the tunable, shape-changing behavior of SU-8 bilayers, we engineered a 3D MEA that establishes evenly distributed points of contact across the entire surface of the organoid, which is essential for capturing chamber-specific drug responses, mapping signal propagation, and identifying arrhythmic foci^36^. This self-folding mechanism is especially promising for use in biological systems as it is activated by the diffusion of aqueous solvents, enabling device actuation in cell media without external micromanipulation. Furthermore, SU-8 is biocompatible and optically transparent, which allows for simultaneous fluorescence imaging of devices fabricated on quartz or glass substrates. For the first time, we demonstrated 3D electrophysiology mapping of whole cardiac organoids. This bio-interfacing 3D MEA presents a promising avenue for expediting the discovery of novel cardiovascular disease treatments and can be extended to study other electrogenic cell models, such as brain organoids and skeletal muscle organoids.

## Results

### Design and fabrication of 16-electrode shell MEA.

**Figure 1.**
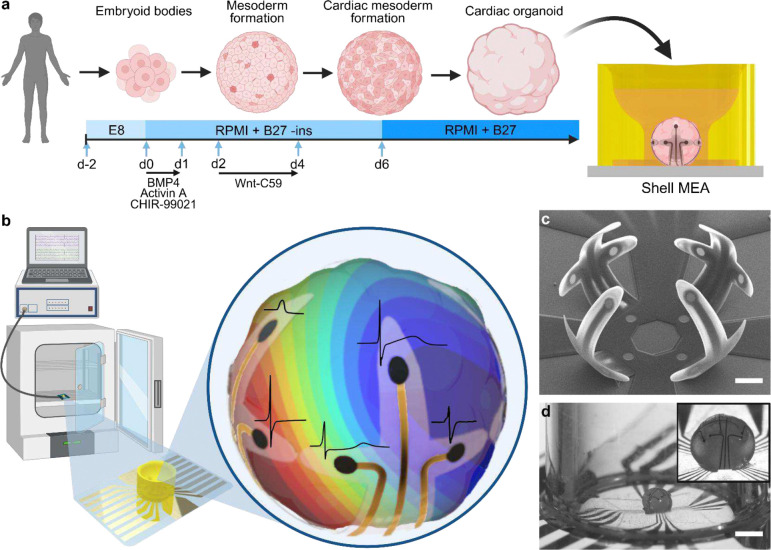
Shell MEA integrated with a 3D-printed microwell for cardiac organoid encapsulation. **(a)** A schematic diagram showing the protocol used to differentiate WTC11 cells into cardiac organoids. **(b)** Conceptualization of the shell MEA system for activation mapping of cardiac organoids with representative waveforms recorded from spontaneously beating cardiac organoids. **(c)** SEM image of an empty folded shell MEA. Scale bar, 100 μm. **(d)** Optical image of a cardiac organoid in a shell MEA taken with a stereomicroscope. The 3D-printed well and media were removed prior to imaging to improve visibility. Inset shows a zoomed-in view of the cardiac organoid encapsulated in the shell MEA. Scale bar, 1 mm. Figures a and b were created with BioRender.com with publication license.

Self-assembling cardiac organoids offer a low-cost and high-throughput model of the human heart by recapitulating early cardiogenesis^11^ (Fig. 1a). However, the inhomogeneous and uncontrolled spatial organization of cells makes it challenging to conduct functional analysis that accounts for the nonuniform architecture of cardiac organoids^7^. A key consideration for the shell MEA design was to achieve evenly spaced electrodes that span the entirety of the 3D organoid while minimizing surface coverage to allow sufficient media and oxygen exchange. The ability to record electrophysiology from all surfaces of the organoid is particularly important for understanding how electrical signals propagate throughout the tissue and for mapping their activation-repolarization patterns as conceptualized in Fig. 1b.

**Figure 2.**
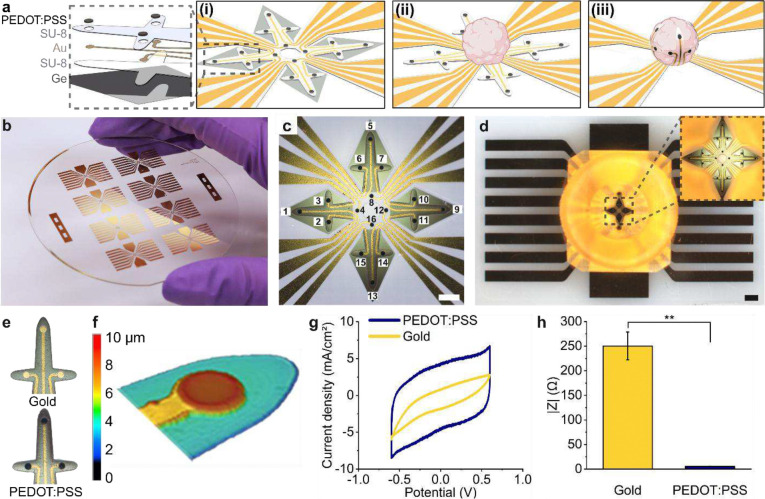
Shell MEA fabrication and electrochemical characterization. **(a)** Schematics showing the step-by-step folding of the shell MEA around an organoid. (i) First, the sacrificial Ge layer is dissolved in H_2_O_2_, and the second spin-coated SU-8 layer is activated by leaching the uncrosslinked monomers of the underexposed regions with acetone. The inset shows an exploded view of the five device layers consisting of Ge, SU-8 2002/2005, Au, partially crosslinked SU-8 2002/2005, and PEDOT:PSS. (ii) After sterilization with ethanol, the organoid is transferred into the microwell that guides the organoid toward the center of the shell MEA. (iii) Once submerged in aqueous media, the device autonomously folds around the organoid over time. **(b)** Optical image of a 3-inch glass wafer with eight shell MEAs. **(c)** Top-view optical image of a fabricated shell MEA showing the electrode configuration. Scale bar, 200 μm. **(d)** Top-view image of a shell MEA device assembled with a microwell. Inset shows a zoomed-in view of the area where the organoid is placed. Scale bar, 1 mm. **(e)** Zoomed-in optical images showing the gold electrodes (top) before and (bottom) after PEDOT:PSS electrodeposition. **(f)** Laser scanning microscope image showing the overall thickness of the PEDOT:PSS coated area (~10 μm). **(g)** Representative cyclic voltammograms of the electrodes in 1X PBS at 100 mV/s before (yellow) and after (blue) PEDOT:PSS electrodeposition (n=4). **(h)** Electrochemical impedance of the electrodes at 1 kHz frequency before (yellow) and after (blue) PEDOT: PSS electrodeposition. Paired *t* test, **P < 0.01; mean ± SEM (n = 4). Figure a was created with BioRender.com with publication license.

We developed a 3D MEA design consisting of four cross-shaped segments that bend into a spherical cage-like configuration (Fig. 1c). In the folded configuration, there are four equidistant electrodes located near the bottom of the organoid, eight equidistant electrodes at the equator of the organoid, and another set of four equidistant electrodes near the top of the organoid (Fig 1d). This design takes advantage of the lateral and longitudinal curvatures that result from the differential strain-based folding behavior of SU-8 bilayers, which allows for close contact between the electrodes and the organoid in all dimensions. Based on the previously reported fabrication process used to create shell MEAs^35^, we developed a 16-electrode organoid-encapsulating shell MEA using five computer-aided design (CAD) photomasks (Fig. S1–3) and five thin-film layers (Fig. 2a).

To streamline the organoid insertion process, we developed a custom 3D microwell to guide the organoid toward the center of the shell MEA without obstructing the folding of the device. We 3D printed the structure using a biocompatible resin that could be fastened to the shell MEA substrate (Fig. S4). The funnel-shaped microwell ensures that the organoid gravitates toward the center of the device after dispensing the organoid immersed in cell culture media with a pipette. We can fabricate up to eight devices on a 3-inch wafer in parallel, and this process can be further scaled up for high-throughput device manufacture (Fig. 2b). The sequence of the 16 electrodes is depicted in Fig. 2c on an unfolded shell MEA. By assembling the shell MEA and microwell platform, we can easily insert the cardiac organoids in the shell MEA in a one-step process without the need for micromanipulators (Fig. 2d). Besides electrophysiological measurements, the shell MEA can secure organoids and fix their orientation while being suspended in media. Such spatial control would enable further applications like precise cell targeting to induce acquired arrhythmias, spatially controlled electrical conditioning and chemical patterning for developing multi-chambered cardiac models, and long-term electrophysiology monitoring and mapping.

To enhance the electrochemical performance of the gold electrodes, we deposited a thin layer of highly conductive polymer, poly(3,4-ethylenedioxythiophene) polystyrene sulfonate (PEDOT:PSS) (Fig. 2e). We characterized the electrochemical properties of the gold electrodes before and after deposition of an approximately 5 μm-thick layer of PEDOT:PSS (Fig 2f). Cyclic voltammograms of the PEDOT:PSS-coated electrodes displayed higher charge storage capacity compared to the non-coated electrodes (Fig. 2g). Electrochemical impedance spectroscopy of the PEDOT:PSS-coated electrodes exhibited a nearly 45-fold decrease in impedance (~6 ohm at 1 kHz in phosphate-buffered saline solution) compared to the non-coated electrodes (~250 ohm at 1 kHz in phosphate-buffered saline solution), demonstrating heightened electrochemical sensitivity (Fig. 2h, Fig. S4).

### Characterization of the self-folded organoid-encapsulating shell MEA.

**Figure 3.**
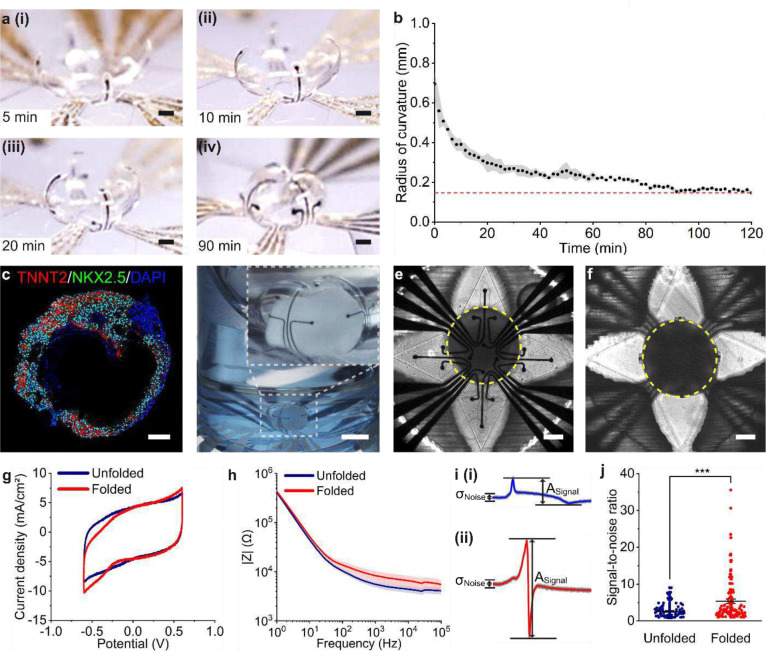
Characterization of folded shell MEA for optimized electrophysiology recording of cardiac organoids. **(a)** Optical images of the shell MEA folding at (i) 5 minutes, (ii) 10 minutes, (iii) 20 minutes, (iv) and 90 minutes after actuation. Water was removed during imaging to reduce distortion. Scale bar, 100 μm **(b)** Radius of curvature of the shell MEA were measured over time after actuation in aqueous media (n=4 devices). **(c)** Immunofluorescence image of a cardiac organoid cryosection stained with TNNT2 (red), NKX2.5 (green), and DAPI (blue). Scale bar, 100 μm. **(d)** Optical image of a cardiac organoid encapsulated in a shell MEA within cell culture medium. Inset shows a zoomed-in view of the electrode arrangement around the cardiac organoid. The microwell was removed before imaging with the stereomicroscope. Scale bar, 1 mm. **(e-f)** Brightfield microscopy images of a cardiac organoid (outlined in yellow) at the center of the shell MEA **(e)** before and **(f)** after device folding. Scale bar, 200 μm. **(g)** Representative cyclic voltammograms of PEDOT:PSS coated electrodes before (blue) and after (red) device folding. **(h)** Electrochemical impedance spectroscopy plots of the PEDOT:PSS-coated electrodes before (blue) and after (red) device folding (n=8 electrodes). **(i)** Representative local field potential waveforms recorded from cardiac organoids in (i) unfolded and (ii) folded shell MEAs. **(j)** Comparison of SNR detected from unfolded and folded shell MEAs showing that device folding significantly improves cardiac organoid electrophysiology recording quality. Paired *t* test, ***P < 0.001; mean ± SEM.

We created shell MEAs for organoids with sizes ranging from 0.5 mm to 1.5 mm in diameter by utilizing different sets of five photomasks (Fig. S1–3). We optimized the folding angle for each dimension by tuning the degree of SU-8 crosslinking, bilayer thickness, and treatment time in acetone (Table S1). Direct contact between the electrodes and cells across the organoid surface provides visualization of the signal propagation across the 3D tissue and improves signal-to-noise ratios for higher-quality recordings^33^. We aimed to program the shape-changing behavior of the SU-8 bilayer to optimize the final folding angle of the device for a known organoid size while providing enough time to insert the organoid and achieve a stable configuration in a biologically relevant time frame. For the chosen SU-8 bilayer thickness, degree of crosslinking, and acetone treatment time, we observed that the device designed for 0.5 mm-diameter organoids began to fold almost immediately after submerging it in aqueous solution (Fig. S5). After 5 minutes, the device self-folded to roughly 31% of its fully folded state (Fig. 3a) and reached the final configuration after 90 minutes, which provided sufficient time to insert the organoid (Fig. 3b). We differentiated self-assembling cardiac organoids from human iPSCs based on an established protocol^5^, and immunofluorescence images of stained cardiac organoid cryosections showed successful differentiation toward cardiomyocytes (Fig. 3c, Fig. S6). By adjusting the cell seeding density, we were able to obtain controlled sizes of cardiac organoids with diameters ranging from 0.5 to 1.5 mm. We inserted the organoids into the shell MEA (Fig. 3d) with a pipette by transferring the organoid in its native media to the microwell. The organoid settled to the bottom of the well located at the center of the shell MEA (Fig. 3e) and we left it in the incubator for at least 1.5 hours for the device to complete folding and allow the organoid to equilibrate to the new environment. Upon visual inspection with a brightfield microscope, we observed beating of the cardiac organoids encapsulated in the shell MEAs (Fig. 3f). Cyclic voltammetry (Fig. 3g) and electrochemical impedance spectroscopy (Fig. 3h) of the PEDOT:PSS-coated electrodes after folding of the device demonstrated no significant changes in the electrochemical performance compared to the unfolded shell MEA. Although the electrochemical sensitivity of the device was not compromised by folding, we observed higher signal quality from the organoids after folding. The representative waveforms recorded from cardiac organoids placed in unfolded and folded shell MEAs are shown in Fig. 3i. The electrophysiology recordings from folded shell MEAs exhibited significantly higher signal-to-noise ratios than electrophysiology recordings from unfolded shell MEAs (Fig. 3j). We find that MEAs capable of establishing conformal contact with organoids achieve superior signal detection compared to planar MEAs.

### Continuous recording and drug response monitoring in cardiac organoids.

**Figure 4.**
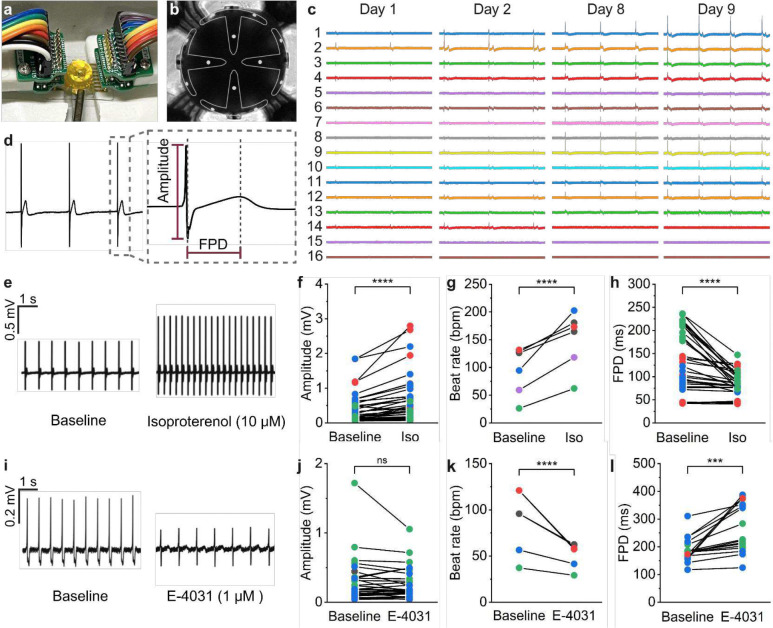
Shell MEA biocompatibility for continuous recordings and drug screening with cardiac organoids. **(a)** Experimental setup of the electrophysiology recording of organoids using shell MEAs. The device is connected to two gold-coated 8-pin clamps and sealed inside a Faraday cage in an incubator with interconnects to the Intan RHS stim/recording system. **(b)** Top-view brightfield microscopy image showing a cardiac organoid encapsulated in the shell MEA with the device outlined in white. **(c)** Electrophysiology recordings of a spontaneously beating cardiac organoid from the 16-electrodes shell MEA recorded on days 1, 2, 8, and 9 after organoid insertion in the device. **(d)** Representative trace from a spontaneously beating cardiac organoid with the waveform parameters, including field potential amplitude and field potential duration, used to characterize the electrophysiological properties. **(e)** Representative traces of cardiac organoids before and after treatment with 10 μM isoproterenol. Changes in **(f)** field potential amplitude, **(g)** beat rate, **(h)** field potential duration after 10 μM isoproterenol (n=5 organoids). **(i)** Example traces of cardiac organoids before and after treatment with 1 μM E-4031. Changes in **(j)** field potential amplitude, **(k)** beat rate, **(l)** field potential duration after 1 μM E-4031 (n=4 organoids). Each color represents drug responses measured from different electrodes surrounding a single cardiac organoid.

Long-term culture is essential for improving the maturity of cardiac organoids to achieve more adult heart-like functionality^37^. A major advantage of shell MEAs is their minimally invasive approach to organoid electrophysiology analysis, enabling continuous *in vitro* monitoring (Fig. S7). Unlike optical imaging techniques that often require additional components or harsh chemicals, shell MEAs allow electrophysiology recordings to be conducted directly in the organoid’s original culture media. During recordings, we housed the shell MEA in a Faraday cage to minimize noise (Fig. S8) and maintained it in a humidified incubator (Fig. 4a). The shell MEA can be easily connected to and disconnected from the recording system without disturbing the cell environment, ensuring sterile conditions for long-term monitoring (Fig. 4b). We tracked the electrophysiology of a spontaneously beating cardiac organoid over nine days and observed region-specific changes from the 16 electrodes among recordings collected on days 1, 2, 8, and 9 (Fig. 4c). We observed a consistent increase in beat rate across days 1, 2, 8, and 9, which suggests enhanced organoid maturity and functionality^38^. Additionally, signal amplitude increased across all 16 electrodes from day 1 to 2, likely reflecting enhanced excitation-contraction coupling among cardiomyocytes^39^. From day 2 onward, most electrodes continued to show increases in amplitude. Several channels exhibited amplitude decreases though, potentially due to the combined effects of technical and biological factors such as increased resistance between the cells and electrodes, degradation of the PEDOT:PSS coating^40^, localized cell stress, or uneven maturation and nutrient diffusion. The sustained activation signals detected nine days post-insertion demonstrate the biocompatibility of the shell MEA for extended studies of cardiac organoids. Moreover, the stable contact between the electrodes and cells at specific regions of the organoid enables long-term monitoring of localized electrophysiological changes. This feature makes shell MEAs particularly valuable for studying electrical pathways in spatially organized, heterogeneous organoids and may offer deeper insights into cardiac development, arrhythmogenesis, and cardiovascular disease pathophysiology.

We investigated the application of shell MEAs for cardiotoxicity screening using cardiac organoids by treating them with several cardioactive components, including isoproterenol and E-4031. We analyzed the electrophysiology recordings from the shell MEAs to assess key parameters such as beat rate, field potential amplitude, and field potential duration (FPD) (Fig 4d). Following treatment with both compounds, we observed significant changes in the waveform. Isoproterenol is a positive chronotropic and inotropic compound used to treat bradycardia conditions^47^. After the addition of 10 μM isoproterenol (Fig. 4e), we observed an approximately 81% increase in field potential amplitude (Fig. 4f), an 84% increase in beat rate (Fig. 4g), and a 20% decrease in FPD (Fig. 4h), findings that are consistent with previously reported data^41,42^. E-4031 is a hERG channel-specific blocker known to prolong atrial and ventricular refractoriness. In line with prior studies^42^, we found that treatment of cardiac organoids with 1 μM E-4031 led to a 12% decrease in field potential amplitude (Fig. 4i), a 34% decrease in beat rate (Fig. 4j), and a 64% increase in FPD (Fig. 4k). Additional drug screenings further demonstrated the ability of the shell MEA to detect changes in electrophysiological changes in the organoid (Fig. S9).

### Local activation time detection for 3D activation mapping.

**Figure 5.**
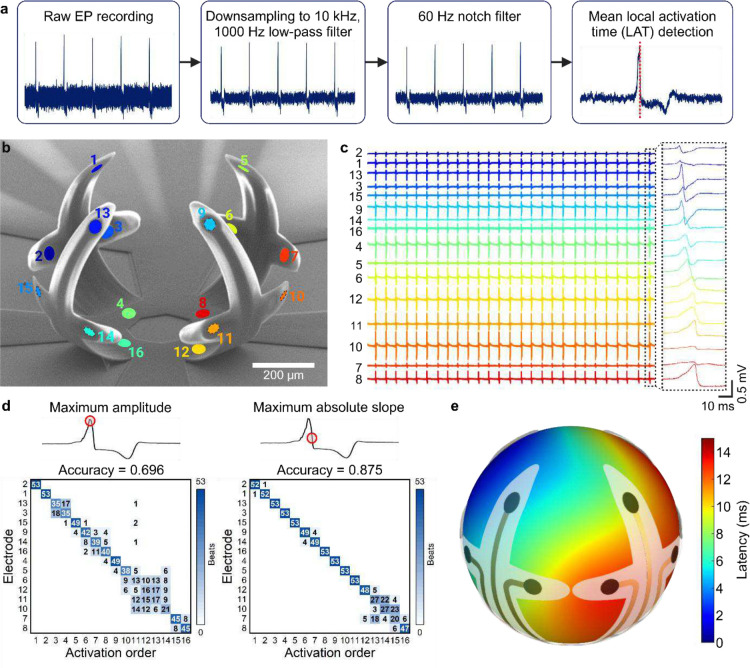
3D Activation mapping of spontaneously beating cardiac organoids. **(a)** Process flow for LAT detection from initial data collection Electrophysiology recordings of spontaneously beating cardiac organoids in shell MEAs are downsampled to 10 kHz. A lowpass filter of 1000 Hz and a notch filter at 60 Hz is applied to minimize interference. Finally, the mean LAT is detected for a series of beats occurring within a 1-minute recording for all 16 electrodes. **(b)** SEM image of an empty folded shell MEA for 500 μm-diameter organoids showing the electrode configuration. **(c)** Representative field potential traces from a cardiac organoid-encapsulating shell MEA. Inset shows a zoomed-in view of the activation latencies between the 16 electrodes. **(d)** Confusion matrices of two LAT detection methods demonstrate that the maximum negative slope-based LAT detection results in greater beating order accuracy than the maximum signal amplitude-based LAT detection for a series of 53 beats detected in a 1-minute recording of cardiac organoid EP. **(e)** 3D activation map interpolated from the 16 LATs detected across the cardiac organoid.

We generated 3D isochrone maps from the shell MEA electrophysiology recordings by assigning spherical coordinates to each electrode based on the known geometry of the organoid and determining the local activation time (LAT). Activation time refers to the point when cardiomyocytes beneath the electrodes depolarize, which corresponds to the upstroke of the action potential. Simultaneous recordings of unipolar electrograms have shown that this point coincides with the steepest down stroke in unipolar waveforms^43^. We identified the LAT for all 16 electrode recordings, which underwent processing to minimize electrical interference (Fig. 5a). Optimizing the filtering process is essential for reducing far-field artifacts while preserving data integrity^35,43^. We found that a 1000 Hz low-pass filter resulted in the greatest activation ordering accuracy for recordings with high SNR without significantly altering the waveform (Fig. S9, S10). For recordings with lower SNR, a 100 Hz low-pass filter was required for improved accuracy. Additionally, we downsampled the 30 kHz electrophysiology recordings to 10 kHz to process the data more efficiently. Depending on the recording quality, we applied a 60 Hz notch filter to remove line-frequency interference. The electrode layout of the shell MEA in its folded configuration is shown in Fig. 5b. The delay in activation time can be observed from the original EP recordings from the 16 electrodes (Fig. 5c), which are ordered from first to last detected activation from top to bottom and indicated in blue to red. We set the first detected activation time as the starting point and computed the latencies for each channel for subsequent activation (Fig. S11). We assigned corresponding colors to the electrodes shown in Figure 5b for visualization of the activation sequence across the shell MEA. For activation mapping, unipolar electrodes are advantageous as they are not dependent on the relative positioning of two electrodes as in the case of bipolar electrodes, and thus can reliably detect LAT regardless of wavefront orientation^15^. Moreover, variations in signal amplitudes caused by differences in cell-electrode distance are less likely to be reflected in the LAT, as it is detected from the point of maximum slope rather than the wave peak. Indeed, LAT determined from the maximum slope resulted in more consistent activation sequencing compared to LAT determined from the maximum amplitude. The activation order of the 16 electrodes derived from the maximum slope-based LAT for 53 consecutive beats in a 1-minute recording had an accuracy of 0.875, whereas the activation order derived from the maximum amplitude-based LAT had an accuracy of 0.696 (Fig. 5d).

The morphology of unipolar waveforms can also reveal important information regarding the position of the electrode in relation to the wavefront. Specifically, the electrode at the origin of activation generates a negative wave because the electrode is always located in the negative region of the extracellular field potential during signal propagation. As the activation front approaches an electrode, the waveform exhibits a positive peak; conversely, as the activation front moves away from the electrode, it exhibits a negative peak, resulting in a biphasic waveform. We see a similar trend in our electrophysiology recording, where the electrode with the first detected LAT exhibits a large negative peak, followed by more biphasic waveforms with large amplitudes in both negative and positive directions, and finally waves with greater positive amplitudes. These findings suggest that waveforms with an initial sharp negative deflection correspond to electrodes near the activation origin. Clinically, such signals indicate sites where normal or abnormal impulse formation occurs and can be used to identify the arrhythmogenic site of focal cardiac arrhythmias or reveal a tissue discontinuity, which can be symptomatic of diseases like Wolff-Parkinson-White (WPW) syndrome and myocardial infarction^43^.

To create the 3D isochrone map, we computed activation gradients based on the latency data acquired from the 16-electrode shell MEA using radial basis function (RBF) interpolation, assuming a spherical shape for the organoid. RBF is useful for creating high-density vector fields and is less prone to errors in LAT than other interpolation methods like triangulation and finite difference^44^. RBF interpolations have been used in clinical settings to identify ectopic foci and wavefront collision^45^. Through evaluation of various shape parameters (Fig. S12), which control the smoothness of the surface, we generated a 3D map showing an overall latency of approximately 15 ms across a 500 μm-diameter organoid (Fig. S13). The resulting propagation pattern appears to align well with the electrode activation sequence shown in Fig. 5b, with a general propagation from left to right in the presented viewing angle (Fig. 5e). This 3D activation mapping method can provide more detailed information regarding the shape of the wavefront, offering significant insights into the tissue microarchitecture and properties of cells in specific areas. For example, convex wavefronts that propagate slower often indicate source-sink mismatch and concave wavefronts that propagate faster may indicate a larger cardiomyocyte to non-excitable cell ratio^45^.

### Comparing propagation patterns with calcium imaging and conduction velocity mapping.

**Figure 6.**
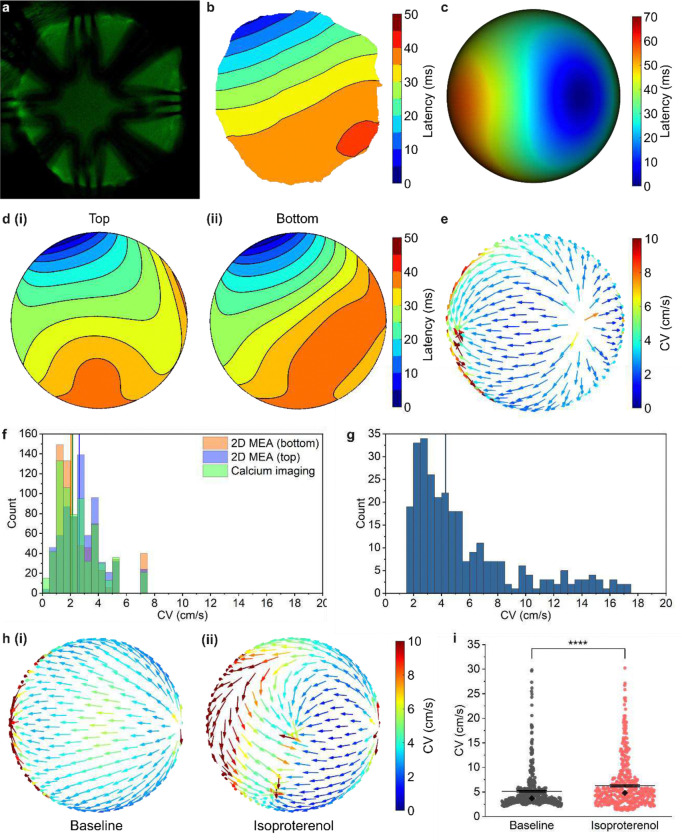
3D conduction velocity mapping and validation with calcium imaging. **(a)** Fluorescence image of a Fluo-4 AM-stained cardiac organoid encapsulated in a shell MEA. **(b)** Isochrone map generated by smoothing a pixel-by-pixel analysis of calcium imaging data. **(c)** 3D isochrone map generated from a shell MEA electrophysiology recording of the cardiac organoid immediately after calcium imaging. **(d)** 2D projections of the (i) upper hemisphere and (ii) lower hemisphere of the 3D isochrone map that reflects the signal propagation in the shell MEA-encapsulated cardiac organoid observed from a viewing angle parallel to that of the fluorescence microscope used for calcium imaging. **(e)** CV vectors generated from the 3D isochrone map reflecting the signal propagation speed and direction across the shell MEA-encapsulated cardiac organoid. **(f)** Histogram showing CV values derived from the isochrone maps generated from calcium imaging and top-view and bottom-view 2D projections of the 3D isochrone map. Vertical lines indicate the median values. **(g)** Histogram showing CV values derived from the 3D isochrone map. Vertical line indicates the median value. **(h)** CV vectors generated from a 3D isochrone map of another shell MEA-encapsulated cardiac organoid reflecting the spontaneous signal propagation speed and direction (i) before and (ii) after treatment with 10 μM isoproterenol. **(i)** Comparison of CV values detected from the shell MEA-encapsulated cardiac organoid before and after isoproterenol treatment.

Unlike conventional MEAs, the transparency of our device enables simultaneous electrophysiology recording and optical imaging. By staining cardiac organoids with calcium indicators, we can directly compare activation maps derived from electrophysiology data with those from calcium imaging (Fig. 6a–b). We recorded the electrophysiology of the same cardiac organoid immediately after calcium imaging and generated a 3D isochrone map (Fig. 6c, S14). However because calcium transient observations are limited to the 2D perspective of a fluorescence microscope^46^, we created 2D projections of the 3D isochrone map for direct comparison. This was done by compiling recordings from 12 electrodes in the upper hemisphere—comprising eight electrodes at the equator and four in the upper region—and 12 electrodes in the lower hemisphere—comprising eight equatorial electrodes and four in the lower region. These data were then assembled to match the viewing angle of the fluorescence microscope at the top and bottom planes (Fig. 6d). We found that both the top and bottom view projections of the 3D isochrone map exhibited strong correlation (>0.94) with the calcium imaging-derived isochrone map. Specifically, the bottom-view map had a mean absolute error of 10.2%, while the top-view map had a slightly higher error of 14.9%. We hypothesize that the bottom-view projection aligned more closely with the calcium imaging data because the inverted microscope primarily captures fluorescence from the organoid’s lower surface, making calcium transients in this region more prominent. Beyond calcium imaging, our shell MEA is compatible with advanced imaging techniques, such as confocal and fluorescence microscopy and laser microdissection for targeted ablation.

Conduction velocity (CV) is defined as the distance traveled by a wavefront per unit of time and provides valuable insights into the initiation and propagation of cardiac activation waves. We calculated the CV from the 3D isochrone map (Fig. 6c) by applying finite difference methods to compute gradients of the interpolated LAT values on the spherical surface. These values were then normalized to obtain the CV vectors, which are mapped on a sphere to show both the speed and direction of the wavefront propagation (Fig. 6e). We also derived CV values from the 2D isochrone maps generated from calcium imaging data (Fig. 6b) and the top-view and bottom-view 2D projections (Fig. 6d) of the 3D isochrone map (Fig. 6f). The mean CVs were 2.54 cm/s, 2.86 cm/s, and 2.63 cm/s for the calcium imaging, top-view 2D MEA, and bottom-view 2D MEA isochrone maps, respectively. As shown in Fig. 6f, the bottom-view 2D MEA map exhibits a slightly higher correlation with the calcium imaging data, likely due to the inverted microscope capturing more fluorescence from the lower plane of the organoid. Notably, the CV values obtained from the 3D isochrone map were higher, with a mean of approximately 5.78 cm/s (Fig. 6g). This discrepancy may arise from the inherent limitations of calcium imaging, including lower temporal resolution and the need for spatiotemporal filtering to remove optical signal processing artifacts. Furthermore, 2D isochrone maps are restricted to a single plane, which overlooks potential faster conduction paths in deeper layers of the organoid. These findings highlight the unique capability of our shell MEA to capture a broader range of electrical activity in 3D cell models. The higher CV observed in the 3D isochrone map likely reflects a more comprehensive assessment of wavefront propagation throughout the entire organoid, rather than a restricted 2D plane. Additionally, the 3D isochrone map considers the circumferential distance traveled by the wavefront, which more accurately accounts for the full tissue area involved in conduction, as opposed to linear distances used in 2D activation maps. While 3D isochrone mapping may involve greater latency interpolation, leading to potential deviations, it provides a more physiologically relevant measurement of conduction dynamics in complex, 3D cardiac structures.

We compared the CV values of a cardiac organoid before and after treatment with 10 μM isoproterenol (Fig. 6h, S15). Following isoproterenol addition, the mean CV increased from 5.12 cm/s to 6.28 cm/s, while the median conduction velocity increased from 3.70 cm/s to 4.82 cm/s (Fig. 6i). This increase in conduction velocity is consistent with the positive dromotropic effects of isoproterenol reported in previous studies^47^. Interestingly, 3D CV maps revealed nonuniform changes in propagation patterns. While activation initially propagated at a similar speed and direction under both conditions, wave propagation accelerated significantly in the latter half of the organoid post-isoproterenol treatment. This demonstrates the advanced capability of our 3D mapping system in detecting region-specific variations in activation patterns, which could revolutionize spatially organized cardiac organoid development for preclinical cardiac research.

## Discussion

In this study, we present a new 3D activation mapping system for cardiac organoids using integrative 16-electrode shell MEAs. Though recent advances in 3D MEAs have demonstrated considerable progress toward flexible and shape-changing devices, our minimally invasive shell MEA offers extensive spatiotemporal information by capturing electrical activity across entire surfaces of cardiac organoids. The optical transparency of our shell MEA provides unique opportunities to conduct simultaneous live imaging for comprehensive functional analysis, which we demonstrate by imaging calcium transients and comparing them to propagation patterns generated from electrophysiology recordings. Additionally, the integrated microwell allows immediate incorporation of organoids with shell MEAs, eliminating the need for external micromanipulators. The on-chip fabrication process and seamless operation of the shell MEAs unlock possibilities for developing large-scale drug screening platforms and multimodal devices integrated with MEMS and microfluidics to monitor organoid microenvironments^48^.

These advancements lay the groundwork for developing more physiologically relevant cardiac organoid models, and show promising applications for studying complex electrophysiological mechanisms, especially in disease models. Recent studies have highlighted the capacity of cardiac organoids to replicate more sophisticated structures of the heart^49^, including distinct atrial and ventricular regions by electrical conditioning^41,50^; multiple chambers by co-developing progenitor subsets^51^; and hypoxic gradients to model myocardial infarction^52^. Such cardiac organoids offer unparalleled possibilities to study tissue-scale and acquired arrhythmias that are beyond the reach of conventional 2D monolayer cultures (Fig. S16). Combined with iPSCs from patients with inherited arrhythmic disorders, these organoids could serve as personalized platforms for disease modeling, drug testing, and precision medicine^5^. Our shell MEAs would enable 3D activation mapping of these models to reveal detailed propagation pathways for accelerating our understanding of complex conduction mechanisms.

Some limitations of our mapping algorithm lie in the fact that the electrophysiology recordings are restricted to 16 points on the organoid, requiring interpolation of activation data for the remaining areas. Depending on the quality of the recordings and interpolation method, the resulting conduction velocity vectors can vary. As such, we often observe outliers, especially near the activation origin and end-point. We can increase the number of electrodes on our shell MEA for 3D activation maps with enhanced spatial resolution and thus more accurate conduction velocity estimations. Additionally, incorporating machine learning algorithms would enable in-depth analysis of conduction pathways and wavefront curvatures to identify specific arrhythmias^53^. Recently, omnipolar electrodes have been explored as an alternative to conventional unipolar and bipolar electrodes for cardiac activation mapping^54^. The vector-based signal reconstruction of omnipolar shell MEAs may allow more accurate and real-time conduction velocity measurements by removing far-field artifacts and directly computing wavefront orientation^53,55^.

Our shell MEA system represents a transformative tool for cardiac research as we demonstrate 3D activation mapping of whole organoids for the first time. By addressing current limitations and leveraging cutting-edge mapping algorithms, we anticipate it will drive innovation in cardiac modeling, disease research, and drug discovery based, ultimately advancing personalized medicine for cardiovascular diseases. This system provides a powerful platform for long-term organoid studies and can be expanded to other electrogenic organoids, enabling precise detection of pharmacological responses and facilitating more accurate predictions of human outcomes.

## Methods

### Organoid-encapsulating shell MEA fabrication

We fabricated the conformal shell MEAs by serial photopatterning. First, we spin-coated positive photoresist S1827 (Kayaku, Westborough MA) at 3000 rpm on 3-inch quartz wafers (NanoSilicon, San Jose CA), and exposed it to UV light at an energy density of 120 mJ/cm^2^ under a photomask designed in AutoCAD to define the Ge pattern. After developing the photoresist in 351 Developer (diluted 1:5 with water, Kayaku, Westborough MA), we deposited 50 nm of Ge via thermal evaporation. We lifted off the photoresist in acetone to obtain the desired Ge pattern. For the shell MEA designed for 500 μm-diameter organoids, we spin-coated a 7:3 mixture of negative photoresists SU-8 2005 (Kayaku, Westborough MA) and SU-8 2002 (Kayaku, Westborough MA) at 3000 rpm. We soft-baked the SU-8 at 65°, 95°, and 65° for 1, 3, and 1 minute respectively. We exposed the SU-8 layer to UV light at an energy density of 240 mJ/cm^2^ under a photomask designed for the bottom electrode insulating layer. We repeated the baking procedure and placed it in SU-8 developer (Kayaku, Westborough MA) for 90 seconds to remove the uncrosslinked monomers. Finally, we hard-baked the SU-8 at 150° for 10 minutes. For the shell MEAs designed for 1.5 mm-diameter organoids, we spin-coated SU-8 2005 at 3000 rpm and exposed it to UV light at an energy density of 300 mJ/cm^2^. Next, we patterned the electrode layer by spin-coating S1827 at 3000 rpm, selectively exposing it to UV light at 120 mJ/cm^2^ under a photomask for the electrodes, and developing in 351 Developer (1:5 in water). We deposited 10 nm of Cr before depositing 50 nm of Au to improve the adhesion to the SU-8 layer. We lifted off the photoresist by submerging the wafer in isopropyl alcohol (IPA) for 24 hours and gently sonicating. Next, we spin-coated the second SU-8 layer by repeating the same procedure as the first SU-8 layer. However, we used two different photomasks to selectively expose the folding areas of the shell MEA at a lower UV density of 120 mJ/cm^2^ for the 500 μm-diameter organoids and 180 mJ/cm^2^ for the 1.5 mm-diameter organoids, and to fully crosslink the non-folding areas of the shell MEA by exposing it to UV light at an energy density of 240 mJ/cm^2^ for the device for 500 μm-diameter organoids and 300 mJ/cm^2^ for the device for 1.5 mm-diameter organoids. We repeated the same post exposure baking procedure and placed it in SU-8 developer for 90 seconds to obtain the top insulating layer.

### Microwell assembly and PEDOT:PSS coating

We designed the microwells in SolidWorks and printed them using BIO resin (Boston Micro Fabrication, Maynard MA) with the microArch S240 3D printer (Boston Micro Fabrication, Maynard MA). The microwells underwent post processing to ensure biocompatibility by treating under UV light (Form Cure FormLabs, Somerville, MA) at 50°C for 2 hours and soaking in IPA for 48 hours. To assemble the shell MEA device, we carefully scored the wafer into individual shell MEAs and bonded the microwell on top of the wafer using Dragon Skin^™^ VERY FAST (Smooth-On, Inc., Macungie, PA). To enhance electrode sensitivity, we electrodeposited poly(3,4-ethylenedioxythiophene) polystyrene sulfonate (PEDOT:PSS) solution. Using a platinum rod as the reference, we connected the electrodes to the VersaSTAT3 Potentiostat Galvanostat (AMETEK Scientific Instruments, USA) and applied a current density of 0.4 μA/cm^2^ for 5 minutes to coat the reference electrode and a current density of 0.2 μA/cm^2^ for 5 minutes to coat 8 electrodes in parallel.

### Organoid-encapsulating shell MEA characterization

In order to quantify the effect of PEDOT:PSS deposition on the electrochemical characteristics of the electrodes, we conducted electrochemical impedance spectroscopy and cyclic voltammetry on both bare gold electrodes and the ones with PEDOT:PSS using a VersaSTAT3 Potentiostat Galvanostat (AMETEK Scientific Instruments, USA) with a three-electrode setup. We used Ag/AgCl and platinum wire as the reference and counter electrodes, respectively. We evaluated the impedance from 1 Hz to 10 kHz in 1xphosphate-buffered saline (PBS; pH 7.4), measuring 10 points per decade. Cyclic voltammetry was carried out with the same setup from −0.6 to 0.6 V at a scan rate of 100 mV/s. Furthermore, we measured these properties before and after folding to assess the effect of the folding*.* We measured the folding of shell MEAs in water at 37°C from time-lapse images taken at 10-second intervals with the MicroTester G2 (CellScale, Ontario, Canada). We calculated the radius of curvature by selecting 3 points on the image and using a custom-made code on Python.

### hiPSCs culture and cardio organoid generation

We maintained human induced pluripotent stem cells (hiPSCs) (WTC11, UCSFi001-A) in Essential 8 medium (E8, Thermo, A1517001) on 1% Geltrex (Corning, A1413202) / DMEM-F12 (Thermo, 11320033) coated six-well plates. We passaged the hiPSCs at 80% to 90% confluency with 0.5μM EDTA/DPBS (Invitrogen, AM9260G) every 3 to 4 days. We differentiated cardio organoids based on a published study^5^ [A]. Briefly, we pipetted 20k hiPSCs suspended in E8 medium supplemented with 10μM Rock inhibitor (Y-27632, Tocris, 1254) into each well of low-attachment 96 well plates to form the embryoid bodies (EBs) at day 0. At day 1, we cultured the EBs with RPMI 1640 (Thermo, 11875093) / 2% B-27^™^ -insulin (Thermo, A1895601) supplemented with 4μM of CHIR99021 (Tocris, 4423), 1.25ng/ml BMP4 (R&D system, 314-BP) and 1ng/ml Activin A (R&D system, 338-AC) for 24 hrs, then replaced it with RPMI 1640/ B-27^™^ -insulin and incubated them for another 24 hrs. At day 3, we changed the medium to RPM1 1640/ B-27^™^ -insulin supplemented with 2 μM of Wnt-C59 (Selleckchem, SS7037) and incubated it for 2 days. At day 5, we changed the medium with RPM1 1640/ B-27^™^ -insulin and incubated it for another 2 days.  From day 7 onward, we replaced the medium every 2 days using RPMI 1640/2% B-27^™^ (Thermo, 17504044).

### Immunochemistry and imaging

We fixed the organoids with 4% Paraformaldehyde (PFA) for 1 hr at 4 C°, rinsed with DPBS, and then incubated them in 30% sucrose/DPBS at 4 C° overnight before transferring them to the OCT compound in a 25×15mm cryomold for flash-frozen using the dry ice/100% ethanol slurry. We sectioned the organoid frozen blocks into 15 to 25μm thicknesses using a cryostat (Leica NX70) and adhered them to microscopic slices. We stored the sections at −80 C° before immunochemistry. We rinsed the cryosections with 0.2% Triton X-100/DPBS three times for 5 mins to wash off the OCT compound, then blocked them with 10% donkey serum for 2 hrs at room temperature (RT). Then we incubated the sections in primary antibodies diluted in 10% donkey serum at 4°C overnight, followed by triple rinsing with DPBS for 10 mins, and incubating them in secondary antibody/DAPI in 10% serum for 2 hrs at RT at dark. Then, we thoroughly rinsed the sections with DPBS three times before mounting them on the Fluromount solution on coverslips. The primary antibodies we used for immunochemistry include rabbit anti-NKX2.5 (1:500, Cell signaling tech, 8792), rabbit anti-HAND1 (1:500, Abcam, ab196622), mouse anti-cTnT (1:500, Bioscience, 565744), and mouse anti-CD31 (1:1000, Abcam, ab9498).  Secondary antibodies include donkey anti-rabbit IgG 488 (1:1000, Thermo, A-21206) and donkey anti-mouse IgG 555 (1:1000, Thermo, A-31570). We collected confocal images using a Leica SP8 inverted confocal microscope with the 20X objective. We used ImageJ to process organoids’ fluorescent images.

### Electrophysiology recording

Prior to organoid encapsulation, we activated the shell MEA to initiate folding. We released the folding segments of the shell MEA from the wafer by dissolving the sacrificial Ge layer in 5% hydrogen peroxide solution. Once the Ge layer was dissolved, we sterilized the device in 70% ethanol solution for 30 minutes in a Class II biosafety cabinet. Finally, we treated the shell MEA with acetone for 5 minutes, rinsed it with cell culture medium, and transferred the cardiac organoid in its original media into the microwell for shell MEA encapsulation. During recording, we kept the organoid-encapsulating shell MEA in a Faraday cage, which was placed in a humidified incubator maintained at 37°C with 5% CO_2_. For drug response recordings, drug solutions were prepared by dissolving the cardioactive compounds in RPMI 1640/ 2% B-27^™^ warmed to 37°C. The spontaneous baseline activity was recorded for 20 minutes before sequentially treating the organoids with increasing concentrations of isoproterenol and E-4031. Recordings were taken at least 20 minutes after drug treatment to provide sufficient time for organoids to equilibrate to the new environment. Data recording was performed with an Intan recording system (Intan Technologies, Los Angeles, CA, USA) at a sampling rate of 30 kHz.

### Local activation time detection

We downsampled 30 kHz electrophysiology recordings to 10 kHz and applied 1000 Hz low-pass and 60 Hz notch filters to remove powerline inference. For recordings exhibiting strong interference, we applied a 100 Hz low-pass filter. We processed the data using custom Python scripts. We identified the local activation times (LAT) by detecting the maximum negative slope of the field potential. We identified individual beats using the Nonlinear Energy Operator (NEO), defined as:

(1) $$NEO[x(n)]=x(n{)}^{2}-x(n-1)\cdot x(n+1)$$

Where x(n) represents the signal at time n.

We determined the thresholds for beat detection on a case-by-case basis. We found the LAT for each beat by detecting the largest negative slope. This slope, $S_{max}$, is defined as:

(2) $$S_{max}=min\left(\frac{dx}{dt}\right)$$

where $\frac{dx}{dt}$ represents the derivative of the signal x with respect to time t. We determined the dt activation order of the 16 electrodes by finding the average LATs of each electrode for all beats detected in a one-minute recording. We then calculated the relative latency for each electrode respective to the first activating electrode to find the average relative latencies. We illustrated the accuracy of the activation order by creating a confusion matrix that captures the frequency with which each electrode appears at a given rank across all detected spikes. We manually reviewed each LAT by overlaying the waveforms to ensure accuracy.

### Electrophysiology waveform analysis

To analyze the electrophysiology of the cardiac organoids, we measured three key parameters—field potential amplitude, beat rate, and field potential duration. We determined the field potential amplitude by measuring voltage differences between the maximum and minimum amplitude of the wave. We calculated the interbeat interval by measuring the time interval between LATs of consecutive beats and used this value to determine the beat rate (bpm). We measured field potential duration (FPD) by finding the time between the LAT and the first repolarization peak, which is characterized by either the maximum or minimum amplitude for a positive or negative peak, respectively. To assess the signal quality, we measured the signal-to-noise ratio (SNR) by separating the signal into signal segments and noise segments. We used the following equation calculate SNR:

(3) $$SNR=\frac{\text{Amplitude}}{\sigma_{\text{noise}}}$$

where Amplitude is defined as the voltage difference between the maximum and minimum potential and $\sigma_{\text{noise}}$ is the standard deviation of the noise segment.

#### 3D activation mapping

We interpolated the LATs on a 3D spherical surface, assuming the shape of an ideal sphere. We used radial basis function (RBF) techniques to interpolate the data, where we calculated the distances using the Haversine formula to account for spherical geometry.

The Haversine formula used to calculate the distance between two points on the sphere is defined as:

(4) $$d=2r\cdot arcsin\left(\sqrt{{sin}^{2}\left(\frac{\Delta \phi }{2}\right)+cos\left(\phi_{1}\right)\cdot cos\left(\phi_{2}\right)\cdot {sin}^{2}\left(\frac{\Delta \lambda }{2}\right)}\right)$$

where $\Delta \phi =\phi_{2}-\phi_{1}$ is the difference in latitude; $\Delta \lambda =\lambda_{2}-\lambda_{1}$ is the difference in longitude; r is the radius of the sphere; $\phi_{1}$ and $\phi_{2}$ are the latitudes of points 1 and 2 (in radians); $\lambda_{1}$ and $\lambda_{2}$ are the longitudes of points 1 and 2 (in radians). The Radial Basis Function (RBF) used for interpolation is defined as inverse quadratic function:

(5) $$RBF(d)=\frac{1}{\left(1\;+\;{\left(\frac{d}{\epsilon }\right)}^{2}\right)}$$

where d is the distance between points, and ϵ is a parameter that controls the width of the RBF. To interpolate LAT values at a certain point, we first calculate the distance vector $d=\left[d_{1},d_{2},\ldots ,d_{N}\right]$ between the new point and all known points using the Haversine formula. We then compute the RBF weights w = RBF(d) using the distance vector d. The value at this point is finally interpolated by:

(6) $${LAT}_{\text{new}\;}=\frac{\sum_{i=1}^{N}w_{i}\;\cdot \;{LAT}_{i}}{\sum_{i=1}^{N}w_{i}}$$

We mapped the interpolated LAT values onto a grid spanning the surface of the sphere. Then we created 3D maps using the Python Visualization Toolkit (VTK, Kitware Inc., Clifton Park, NY, USA).

#### 3D CV mapping

We calculated conduction velocity (CV) from gradients of the interpolated LAT values on the spherical surface. Specifically, we used finite difference methods to compute these gradients, which were then normalized to obtain the CV vectors. The resulting CV map was visualized to illustrate the direction and speed of electrical propagation across the sphere, using the Python Visualization Toolkit (VTK, Kitware Inc., Clifton Park, NY, USA).

To perform the finite difference method, we first calculate the gradient in x-direction (∇x) and y-direction (∇y):

(7) $$\nabla x=\frac{{LAT}_{\text{east}}\;-\;{LAT}_{\text{west}}}{2\;\cdot \;\Delta d}\;\nabla y=\frac{{LAT}_{\text{north}}\;-\;{LAT}_{\text{south}}}{2\;\cdot \;\Delta d}$$

We then normalized the gradients to obtain the unit vectors indicating the direction of conduction velocity:

(8) $$\begin{array}{c} {CV}_{x}=\frac{\nabla x}{\sqrt{\nabla x^{2}+\nabla y^{2}}},\\ {CV}_{y}=\frac{\nabla y}{\sqrt{\nabla x^{2}\;+\;\nabla y^{2}}} \end{array}$$

### Calcium imaging data processing

We stained the shell MEA-encapsulated cardiac organoid with calcium indicator Cal-520 AM (AAT Bioquest, 21130) for one hour in a humidified incubator. Prior to imaging, we replaced the solution with Hanks’ Balanced Salt Solution (Thermo, 14025092) and incubated it for 10 minutes. We recorded a fast time-lapse of the cardiac organoid with the Nikon ECLIPSE Ti (Nikon, Tokyo, Japan) at 60 Hz. We analyzed each frame using a custom-made Python code. The position of the electrodes was estimated based on the dimension of the device visible in the recording. To create an isochrone map from the calcium imaging data, we applied a custom mask to exclude background noise and areas covered by the gold electrodes of the shell MEA. The intensity was calculated as the mean intensity of the pixel and its surrounding pixels. The LAT for each pixel was defined as the time at which the signal reached half-maximum intensity. Max pooling with a 5×5 window was applied to the LAT array to reduce the computational complexity. For each pixel in the pooled data, we fitted a 2D polynomial to its local neighborhood:

(9) $$T(x,y)=a+b\cdot x+c\cdot y+d\cdot x^{2}+e\cdot x\cdot y+f\cdot y^{2}$$

where T(x,y) is the fitted activation time, and (x,y) are local coordinates of the pixels. We used least squares optimization to determine the coefficients a,b,c,d,e, and f in the polynomial fitting process. The window size for polynomial fitting was set to 600 μm, corresponding to approximately 149 pixels in the pooled data. This window size matched the organoid radius, ensuring a smooth LAT representation.

We calculated CVs from the calcium imaging-based isochrone map by taking the partial derivatives on the fitted plane. For a given point $\left(x_{0},y_{0}\right)$, the gradient in the x-direction (∇x) is computed as:

(10) $$\nabla x=\frac{T\left(x_{0}\;+\;1,\;y_{0}\right)\;-\;T\left(x_{0}\;-\;1,\;y_{0}\right)}{2\;\cdot \;\Delta \text{pixel}}$$

The gradient in the y-direction (∇y) is computed as:

(11) $$\nabla y=\frac{T\left(x_{0},\;y_{0}\;+\;1\right)\;-\;T\left(x_{0},\;y_{0}\;-\;1\right)}{2\;\cdot \;\Delta \text{pixel}}$$

where Δpixel is the distance per pixel. The gradients are then normalized to obtain the unit vectors indicating the direction of conduction velocity from the previously mentioned formula (10).

Comparison of 2D shell MEA projections with calcium imaging-based isochrone mapping We performed a side-by-side comparison of the 2D projections of 3D shell MEA data with the calcium imaging-based isochrone mapping. We used the Python Visualization Toolkit used to perform 2D projections of the 3D isochrone map. We captured high-resolution screenshots of the 3D isochrone map parallel to the desired projection angle without ambient light and shadows.

The projection values are then retrieved from the screenshots through pixel-wise conversion using a custom Python script to create isochrone maps of the 2D projections. The 2D projections were scaled to match the processed calcium imaging isochronal map in pixel dimensions. Points that exist on both the 2D projection and the calcium isochronal map are selected to compare LAT pairwise with Pearson Correlation and mean absolute error. We used the same method for calculating the CVs from the 2D projection isochrone maps to find the CVs from the calcium imaging-based isochrone map and compared CVs at coexistent points for comparison.
